# Smoking cessation and shared decision‐making practices about lung cancer screening among primary care providers

**DOI:** 10.1002/cam4.3714

**Published:** 2021-01-18

**Authors:** Maria A. Lopez‐Olivo, Jennifer A. Minnix, James G. Fox, Shawn P. E. Nishi, Lisa M. Lowenstein, Kristin G. Maki, Viola B. Leal, Ya‐Chen Tina Shih, Paul M. Cinciripini, Robert J. Volk

**Affiliations:** ^1^ Department of Health Services Research The University of Texas MD Anderson Cancer Center Houston TX USA; ^2^ Department of Behavioral Science The University of Texas MD Anderson Cancer Center Houston TX USA; ^3^ Division of Pulmonary & Critical Care Medicine The University of Texas Health East Texas Tyler TX USA; ^4^ Division of Pulmonary & Critical Care Medicine The University of Texas Medical Branch Galveston TX USA

**Keywords:** lung cancer screening, primary care providers, shared decision‐making, smoking cessation, survey

## Abstract

**Objective:**

We describe primary care providers’ current practice patterns related to smoking cessation counseling and lung cancer screening (LCS).

**Methods:**

Family, internal medicine, and pulmonary medicine providers from two medical centers were asked to complete an electronic survey to report their practice patterns.

**Results:**

Of 52 participating providers, most reported initiating three major components of a smoking cessation intervention often or very often: advise to quit (50, 96%), assess willingness to quit (47, 90%), and assist with counseling or pharmacotherapy (49, 94%). However, other components were less commonly initiated such as arranging follow‐ups (only 11 providers indicated recommending them often or very often, 21%) and less than half of providers reported that they often or very often recommend cessation counseling or pharmacotherapy of any type (except varenicline), though family medicine providers were more likely to recommend pharmacotherapy compared to the other specialists (*p* < 0.01). The majority of providers (47, 92%) reported that they engage in informed/shared decision‐making about LCS, although only about one‐third (17, 33%) indicated using a patient decision aid. Pulmonary medicine providers were more likely to use decision aids than providers from internal or family medicine (*p* < 0.04).

**Conclusions:**

Within the context of LCS, primary care providers report often having conversations about smoking cessation with their patients who smoke, have no clear preference for type of treatment, and rarely use follow‐up calls or visits pertaining to quitting smoking. While many providers report engaging in shared decision‐making about LCS, few use a decision aid for this conversation.

## Graphical abstract

The purpose of this study was to compare current practice patterns related to smoking cessation counseling and screening for lung cancer among family, internal and pulmonary medicine providers. Our findings indicate differences in lung cancer screening practice based on specialty, suggesting interventions to increase the fidelity of key lung cancer screening components may need to target specific provider types.

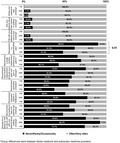

## 1 INTRODUCTION

The estimated number of new cases of lung and bronchus cancer in the United States was 228,820 in 2020, equivalent to 627 lung cancers diagnosed per day.^1^ Lung cancer is the second most common cancer diagnosis by gender, behind prostate cancer for men and breast cancer for women.^1^ The number of estimated deaths due to lung cancer in 2020 is 135,720, representing about 22% of all cancer deaths.^1^

The U.S. Preventive Services Task Force recommends annual lung cancer screening (LCS) with low‐dose computed tomography (LDCT) in high‐risk individuals.^2^ Over 12,000 lung cancer deaths might be prevented every year if heavy smokers 55–74 years of age were screened annually.^3^ Most professional organizations and insurers for LCS (including the Centers for Medicare & Medicaid Services (CMS)), require patient counseling about smoking cessation and shared decision about LCS supported by the use of decision aids.^4^, ^5^, ^6^

Guidance on how best to implement effective strategies for smoking cessation counseling and shared decision‐making for LCS is only beginning to emerge.^7^ Thus, there is wide variation in LCS practice among primary care providers (PCPs) with respect to delivery of guideline‐based smoking cessation treatment and shared decision‐making.^8^, ^9^, ^10^, ^11^, ^12^, ^13^, ^14^ The purpose of this study was to determine PCPs’ current self‐reported practices related to smoking cessation and shared decision‐making for LCS.

## 2 METHODS

The manuscript was prepared according to the strengthening the reporting of observational studies in epidemiology statement.^15^

### 2.1 Study design and setting

This was a cross‐sectional study. Primary care providers from two medical centers were asked to complete a brief, confidential electronic survey to better understand their smoking cessation and LCS practice patterns. The University of Texas Medical Branch (UTMB) is a public academic health science center in Southeast Texas and a designated center for Lung Cancer Screening by the American College of Radiology. Their LCS program is offered to PCPs who wish to refer their patients for shared decision‐making visits, ordering of LCS test, and follow‐up. In 2017, over a 10‐month period 2,781 patients, 55–77 years of age, were identified as current smokers and eligible for LCS. The University of Texas Health Sciences Center at Tyler (UTHSCT) is a public academic health science center in Northeast Texas. In 2012, over a 12‐month period 482 patients, 55–77 years of age, were identified as current smokers and eligible for LCS.

### 2.2 Procedures

We used a snowball approach to recruit participants. Two study collaborators distributed an anonymous electronic survey link via email to PCPs at their institutions from August to November 2019. The survey was distributed via Qualtrics (Qualtrics, Provo, UT). PCPs initially identified then invited other colleagues to complete the survey. The institutional review board of The University of Texas MD Anderson Cancer Center approved this study. A consent statement was provided to participants prior to survey completion.

### 2.3 Participants

Eligible respondents were family physicians, internal medicine physicians, advanced practice providers (i.e., nurse practitioners and physician assistants), and pulmonary medicine providers from the two medical centers that were surveyed. Providers who were retired, or with no patient care responsibilities were excluded. Up to three reminders were sent to participants to encourage survey participation.

### 2.4 Variables

Questions in the survey addressed characteristics of the respondents (gender, specialty, year of graduation from residency training, practice location, and amount of time spent in outpatient care per week). LCS questions included: (a) percent of patients eligible for LCS (i.e., 55–77 years of age, have at least a 30 pack‐year smoking history, currently smoke or quit within the past 15 years, and are appropriate candidates for surgery), (b) the percent of current smokers eligible for LCS, and (c) the respondent's current LCS practice patterns (i.e., whether respondent was referring patients for LCS, had a protocol for LCS, engage patients in shared decision‐making, use of decision aids, and familiarity with Medicare coverage requirements for LCS). The practice patterns items had “yes” or “no” responses except for the familiarity item where the possible responses were “very familiar,” “somewhat familiar,” and “not familiar.”

We also assessed self‐reported performance of 4 of the 5A’s brief intervention model: ask about smoking, advise cessation, assess level of readiness to quit, assist with motivation, or a plan to quit (including use of tobacco cessation medication and counseling), and arrange follow‐up appointment to review progress and adjust the plan.^16^ Though we did not assess the providers’ self‐reported performance of the first step, “ask about smoking,” the electronic health records in both medical centers track patient smoking status and the information is readily available to the providers (this question is updated at every clinic visit; e.g., if a patient has two appointments the same week then they will be asked about smoking at both visits). In addition to the smoking cessation activity questions, we also assessed specific cessation treatment recommendations (i.e., recommend no pharmacotherapy, recommend a single nicotine replacement product, recommend dual nicotine replacement products, recommend pharmacotherapy, and recommend counseling to help patients quit smoking). Possible responses for the smoking cessation activity frequency items were “never,” “rarely,” “occasionally,” “often,” and “very often.”

### 2.5 Data sources

Questions were taken from prior surveys conducted in 2014, administered to providers attending Texas Academy of Family Physicians events in Houston, Dallas, and Austin, Texas, and the Cardinal Health Specialty Solutions Oncology Summit in Coral Gables, Florida.^13^

### 2.6 Analyses

We used descriptive statistics to determine current LCS practice patterns. We reported differences by specialty, which was grouped into three categories: family medicine combined with family nurse practitioners, general internal medicine combined with geriatrics, and pulmonary medicine combined with acute care nurse practitioner in pulmonary medicine. Given that family nurse and acute care nurse practitioners can practice in different fields, we performed a sensitivity analysis by removing these types of providers from the analysis. One‐way analysis of variance (ANOVA) was conducted to compare continuous variables across groups. Chi‐squared tests and Fisher's exact tests, when appropriate, were used to assess the relationship between the categorical variables and provider subgroups. The LCS and smoking cessation services data were analyzed based on completers. For surveys completed twice by the same respondent, we kept the latest record. Year of graduation from residency training was recorded by decade and a separate category was created for providers who were still in training. When responses to continuous variables were provided as a range, we used the median value within the lower and upper limits (e.g., for a range of 1–2, we used 1.5). For the percent of smokers that were eligible for LCS, when number of patients was provided, we calculated the percentages out of the total number of patients eligible for LCS. Results were analyzed both at a level of significance two‐sided α of 0.05, and with a Bonferroni correction to control for multiple comparison testing. All statistical analyses were performed using Stata 15 statistical software (StataCorp LLC).

## 3 RESULTS

### 3.1 Participants

Initially, 33 providers identified by directors of LCS programs at the participating sites (SPN and JF) received the link to participate in the survey by the study investigators. Then, these providers further invited other colleagues. Finally, there were 68 responders, but only 52 unique completed surveys were included in the analysis.

### 3.2 Provider Characteristics

The characteristics of the respondents are shown in Table 1. From the overall cohort, more than two‐thirds of the providers (37/52) were either recent graduates or trainees. Approximately two‐thirds of the family medicine (65%, 13/20) and pulmonary medicine (68%, 13/19) specialists graduated recently (between 2010 and 2019). Eight providers (15%, 8/52) were trainees (six general internal medicine residents and two fellows of pulmonary medicine).

**TABLE 1:** Characteristics of providers across specialties

	Overall cohort (n = 52)	Family medicine (n = 20)	General internal medicine (n = 13)	Pulmonary medicine (n = 19)	*p*‐value*
Gender					
Female	24 (46.2%)	12 (60.0%)	6 (46.2%)	6 (31.6%)	0.20
Graduation year					
<1990	3 (5.8%)	2 (10.0%)	1 (7.7%)	0	**0.009** ^b^
1990–1999	6 (11.5%)	2 (10.0%)	1 (7.7%)	3 (15.8%)	
2000–2009	6 (11.5%)	3 (15.0%)	2 (15.4%)	1 (5.3%)	
2010–2019	29 (55.8%)	13 (65.0%)	3 (23.1%)	13 (68.4%)	
Trainee	8 (15.4%)	0	6 (46.2%)	2 (10.5%)	
Half‐day clinics per week, mean (SD)	4.5 (2.8)	6.5 (2.6)	3.1 (2.7)	3.5 (2.0)	**0.002** ^c^
Average number of patients per half days, mean (SD)	8.0 (2.9)	9.2 (2.5)	6.4 (2.9)	8.0 (2.8)	**0.02** ^c^
Patients eligible for LCS seen in a typical month, mean (SD)^a^	13.9 (14.9)	15.5 (10.0)	11.0 (13.6)	14.1 (20.1)	0.73
Of those, percentage of current smokers, mean (SD)^a^	56.5 (25.2)	62.6 (18.3)	69.2 (21.6)	41.3 (27.6)	**0.003** ^c^
Currently referring patients for LCS	50 (96.1%)	20 (100%)	11 (84.6%)	19 (100%)	0.06
Protocol for identifying patients eligible for LCS	33 (63.5%)	11 (55.0%)	9 (69.2%)	13 (68.4%)	0.61
Engage patients in shared decision‐making about LCS	47 (92.2%)	19 (95.0%)	11 (91.7%)	17 (89.5%)	0.81
Use of PDA about LCS	17 (32.7%)	3 (15.0%)	4 (30.8%)	10 (52.6%)	**0.04** ^c^
Familiarity with Medicare coverage requirements for LCS					
Not familiar	9 (17.3%)	2 (10.0%)	4 (30.8%)	3 (15.8%)	0.50
Somewhat familiar	23 (44.2%)	10 (50.0%)	6 (46.2%)	7 (36.8%)	
Very familiar	20 (38.5%)	8 (40.0%)	3 (23.1%)	9 (47.4%)	

Percentages may not add up to 100% owing to rounding.

Abbreviations: LCS, lung cancer screening; PDA, patient decision aid; SD, standard deviation.

^a^ 2 observations missing.

^b^ This was not statistically significant after applying the Bonferroni procedure to correct for the number of comparisons made.

^c^ Group differences were: Half‐day clinics per week (FM vs. PM 0.001, FM vs. GIM 0.001, GIM vs. PM non‐significant); average number of patients per half days (FM vs. PM non‐significant, FM vs. GIM 0.02, GIM vs. PM non‐significant); percentage of current smokers (FM vs. PM 0.002, FM vs. GIM non‐significant, GIM vs. PM 0.006); use of PDA about LCS (FM vs. PM 0.04, FM vs. GIM non‐significant, GIM vs. PM non‐significant); recommend no pharmacotherapy (FM vs. PM 0.01, FM vs. GIM non‐significant, GIM vs. PM non‐significant).

* Chi‐squared and Fisher's exact tests were used to compare categorical variables or ANOVA for continuous variables.

Gender distribution was similar across specialties. The mean number of half days per week and mean number of patients eligible for LCS seen in a typical month were lower for the general internal medicine providers. When we used the Bonferroni procedure to correct for multiple comparisons, the observed differences were maintained after the post hoc analysis, with the exception of graduation year.

### 3.3 Use of smoking cessation interventions

**FIGURE 1:**
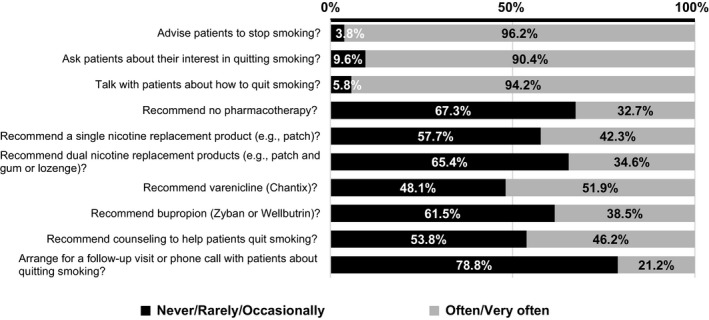
How often primary care providers use smoking cessation interventions with their patients who smoke

**FIGURE 2:**
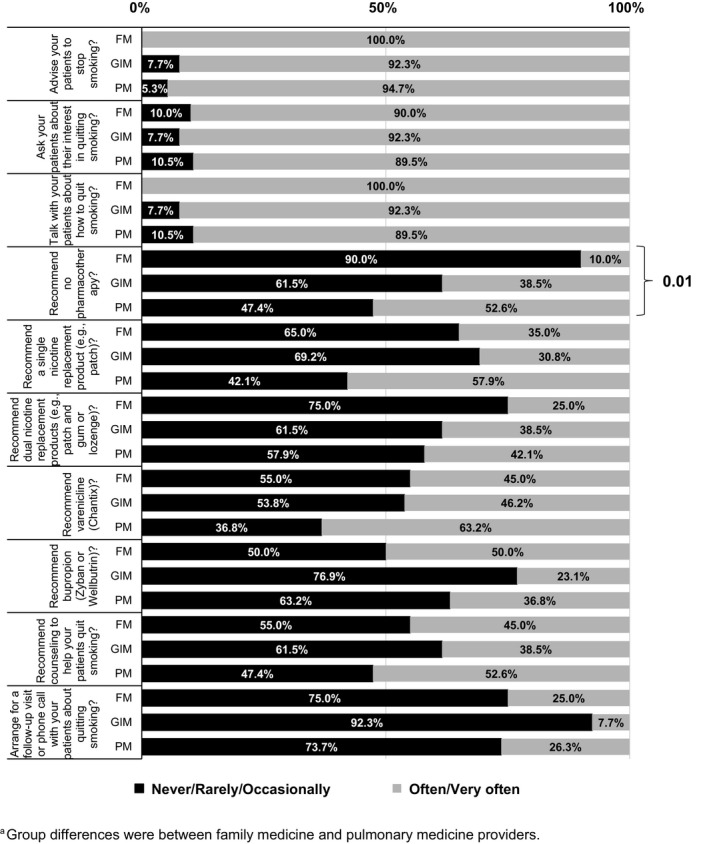
How often primary care providers use smoking cessation interventions with their patients who smoke, per specialty. ^a^Group differences were between family medicine and pulmonary medicine providers

Figure 1 shows the frequency in which providers offer different options to their patients who smoke. In the entire cohort, most providers reported that they often or very often advise patients to stop smoking (96%, 50/52), ask patients about their interest in quitting smoking (assess; 90%, 47/52), and talk with patients about how to quit smoking (assist; 94%, 49/52), though far less report arranging a follow‐up (21%, 11/52). Less than half of providers reported that they often or very often recommend cessation counseling (46%, 24/52) or pharmacotherapy of any type (except varenicline), including a single nicotine replacement therapy (NRT) product (42%, 22/52), dual NRT (35%, 18/52), or bupropion (39%, 20/52), though 52% (27/52) often/very often recommend varenicline. Table S1 shows the comparison of the frequency, by specialty, in which providers offer different options to patients who smoke. No differences across specialty were observed in the providers’ practice related to smoking cessation except for the use of pharmacotherapy, with family medicine providers recommending no pharmacotherapy less frequently than the other specialists (*p* < 0.01) (Figure 2).

### 3.4 Use of shared decision‐making

Most providers (92%, 48/52) reported to engage patients in informed/shared decision‐making about LCS. However, only 33% (17/52) reported the use of a patient decision aid about LCS with their patients. Across specialties, providers reported similar rates regarding having a protocol for identifying patients eligible for LCS, engaging patients in informed/shared decision‐making about LCS, and familiarity with Medicare coverage requirements for LCS. However, using a patient decision aid about LCS with their patients was reported less frequently among family medicine providers (3/20, 15%) compared with other provider types (4/13, 31% for internal medicine and 10/19, 53% for pulmonary medicine providers).

### 3.5 Sensitivity analysis

After removing observations from four providers who could practice in different fields (family nurse and acute care nurse practitioners), we did not observe any changes in our results.

## 4 DISCUSSION

In this descriptive and exploratory study, we assessed the current practices related to smoking cessation and shared decision‐making about LCS among providers of three different medical specialties (i.e., family medicine, general internal medicine, and pulmonary medicine). Compared to the other two specialties, we found that family medicine providers were significantly more likely to actively recommend their patients to use pharmacotherapy for smoking cessation. This difference could be multifactorial; for example, the cost of medications often can be prohibitive. Family medicine clinics have nearly all their patients 100% insured, whereas general internal medicine and pulmonary medicine clinics may not have a high percentage of insured patients or many are covered by Medicare/Medicaid (which has more eligibility restrictions and disqualifications). There is also the possibility that family medicine providers may be more familiar with, or comfortable, discussing pharmacotherapy options, and not recommending pharmacotherapy, than the specialists in general internal medicine and pulmonary medicine. It is also possible that patients seen in the general internal medicine and pulmonary medicine clinics had more comorbidities that prohibited the use of pharmacotherapy or systematically differed in some other way. In addition, there is a negative connotation of pharmacotherapy for smoking cessation, patients from general internal medicine and pulmonary medicine clinics may not be ready to quit and so use of pharmacotherapy is not indicated. Varenicline was recommended most often by providers and dual NRT was recommended the least, though the two treatments have demonstrated similar efficacy for cessation.^17^ Provider performance of the 5A cessation strategies was generally comparable to previous studies.^18^, ^19^, ^20^

The potential harms associated with LDCT include false positive results, radiation exposure, and a potential for overdiagnosis of lung cancer.^21^ The Centers for Medicare & Medicaid Services (CMS) includes LCS with LDCT as a covered service for high‐risk smokers between ages 55 and 77. For screening to be reimbursed, CMS requires a patient counseling and shared decision‐making visit with a qualified health‐care provider prior to referral for screening.^4^ The visit should include counseling about smoking cessation, and patients should be given the opportunity to make a shared decision about LCS supported by the use of decision aids, considering what is important to them related to the potential benefits and harms of screening.^4^, ^5^, ^6^ Here, we found that while most participants reported engaging patients in shared decision‐making about LCS, over two‐thirds of the cohort failed to use patient decision aids; 15% of family medicine providers in our sample used them compared to 53% of pulmonary medicine providers. Evidence suggests provider education through use of decision aids, which are intended to inform patients to prepare them for decision‐making, can also improve provider knowledge.^12^

Most previous studies have focused on either LCS or smoking cessation alone and/or were published before the CMS mandated that a patient counseling and shared decision‐making visit was required for reimbursement of LDCT for LCS.^22^, ^23^ Our data provide more current estimates of providers’ LCS practice and were collected several years after the CMS LCS coverage memo in early 2015.^4^ The study by Triplette et al. surveyed primary care and pulmonary providers within the University of Washington medical system in Seattle, Washington in 2016 about LCS knowledge and program implementation but not smoking cessation options.^12^ They identified gaps in knowledge about key elements of LCS, including knowledge of CMS‐required documentation, patient eligibility, and patient risk after screening; gaps were often specialty specific.

Several studies have published estimates of 5A’s delivery reported by providers. Many of these studies include a broad range of health‐care providers (e.g., midwifes, dentists, psychologists, and pediatricians), report data on specific groups (e.g., pregnant women, African‐American men, medical doctors who are smokers, working age smokers, Medicaid‐enrolled smoker, and veterans), and report on medical training of 5A’s or evaluation of interventions in a controlled setting.^18^, ^24^, ^25^, ^26^ In general, studies report that providers typically perform the “Ask” and “Advise” steps at high rates, less frequently “Assess readiness” to quit or “Assist” with cessation, and rarely “Arrange follow‐up”.^18^, ^19^, ^20^ Providers in our study report similar patterns of delivering the 5A’s to patients who are eligible for LCS compared to self‐reported rates from providers in other clinical contexts (though the delivery of the assist step is higher in our study than typically reported),^18^, ^24^, ^25^, ^26^ and were delivered at higher rates than patient‐reported receipt of the 5A’s in the National Lung Screening Trial (NLST).^27^ Receipt of all 5A’s predicts engagement in cessation counseling and/or pharmacotherapy,^19^ and more frequent delivery of the assist and arrange steps has been associated with increased cessation rates in LCS patients,^20^ suggesting that strategies designed to increase their delivery may considerably benefit patients. Similarly, providers may benefit from education regarding the efficacy, safety, and accessibility of various cessation options and strategies available to their patients.

Compared to our previous findings using a similar survey in 2014 in which approximately 44% of primary care providers mainly based in Texas engaged patients in shared decision‐making for LCS,^13^ the results of the current study indicate better engagement of patients in shared decision‐making with 95% of respondents in family medicine answered affirmative to the shared decision‐making item. Also promising are the data reflecting increased referral of patients to LCS programs: 96.1% of the total cohort reported referring patients for LCS compared to 43.1% of respondents in the 2014 study. While the increases in shared decision‐making about and referral for LCS are encouraging, this likely reflects the difference in the mix of survey respondents as our current survey largely included providers at medical centers, whereas the 2014 survey covered a wider variation of practice settings. More research is needed to determine the robustness of the trends observed.

Patient decision aids for LCS have been shown to increase patient knowledge about lung cancer and the potential harms and benefits of screening, and make patients feel more informed and prepared to make LCS decisions.^28^, ^29^, ^30^ A striking 85% of respondents who identified as family medicine providers did not use LCS decision aids, compared to 47.4% of pulmonary medicine providers. As the decision to commit to annual LCS is complex and multifactorial, tools including decision aids facilitate the decision‐making process; the requirement of the use of decision aids for LCS for CMS reimbursement substantiates their value. Yet, time constraints are a well‐known barrier to providing cancer screenings, and there is an acute need for feasible decision aids for use at the point‐of‐care in clinical practice.

There are limitations to this study. The study was limited to PCPs in Texas, and it is unknown how current practice might differ based on region of the United States. Also, participants were recruited from two medical centers and recruitment from a single health‐care system may further limit generalizability, especially in community settings. Since the survey contained closed‐ended questions, there were limited opportunities for respondents to convey unique responses or explanations of answers. We collected self‐report evidence from providers alone and evidence suggests that delivery of 5A’s may be lower when reported by patients or when documented in the medical record.^18^, ^19^, ^31^, ^32^, ^33^ Our analysis is descriptive and exploratory in nature and as such our results should be interpreted cautiously. Comparisons by specialty may be confounded by other differences in these groups. Finally, we do not have data on factors that may explain survey responses (i.e., low use of patient decision aids or low rates of pharmacotherapy prescription other than varenicline). Future studies should also explore if the use of decision aids or pharmacotherapy prescription rates are associated with lung cancer rates and related mortality.

In summary, while providers in our cohort engage patients in shared decision‐making about LCS, use of patient decision aids is suboptimal. Smoking cessation conversations are prevalent with no clear treatment preference for providers; follow‐up calls or visits pertaining to quitting smoking rarely occur. Our findings indicate differences in LCS practice based on specialty, suggesting interventions to increase the fidelity of key LCS components may need to target specific provider types.

## AUTHOR CONTRIBUTIONS

Study concept and design: Jennifer A. Minnix, Paul M. Cinciripini, Robert J. Volk. Acquisition of data: Maria A. Lopez‐Olivo, James G. Fox, Shawn P.E. Nishi, Viola B. Leal. Analysis and interpretation of data: Maria A. Lopez‐Olivo, Jennifer A. Minnix, Paul M. Cinciripini, Robert J. Volk, Lisa M. Lowenstein, Kristin G. Maki, Ya‐Chen Tina Shih. Drafting of the manuscript: Maria A. Lopez‐Olivo, Jennifer A. Minnix, Lisa M. Lowenstein, Kristin G. Maki, Ya‐Chen Tina Shih, James G. Fox, Shawn P.E. Nishi, Viola B. Leal, Paul M. Cinciripini, Robert J. Volk. Given final approval of the version to be published: Maria A. Lopez‐Olivo, Jennifer A. Minnix, Lisa M. Lowenstein, Kristin G. Maki, Ya‐Chen Tina Shih, James G. Fox, Shawn P.E. Nishi, Viola B. Leal, Paul M. Cinciripini, Robert J. Volk. All authors have agreed to be accountable for all aspects of the work in ensuring that questions related to the accuracy or integrity of any part of the work are appropriately investigated and resolved.

## Supporting information

Table S1

## Data Availability

The data that support the findings of this study are available from the corresponding author upon reasonable request.
