# Supplementary material for: Smoking cessation and shared decision‐making practices about lung cancer screening among primary care providers
Source: Cancer Med. 2021 Jan 18;10(4):1357–65. doi: 10.1002/cam4.3714 (PMC7926026; doi:10.1002/cam4.3714)
Supplement: Supplementary file 1 — Table S1 [file CAM4-10-1357-s001.docx]

**Supplementary Table 1.** How often primary care providers use smoking cessation interventions with their patients who smoke, per specialty

|  | **Family Medicine**  **(n=20)** | **General Internal Medicine**  **(n=13)** | **Pulmonary Medicine**  **(n=19)** |
| --- | --- | --- | --- |
| **Advise your patients to stop smoking?** |  |  |  |
| Never/Rarely/Occasionally | 0 | 1 (7.7%) | 1 (5.3%) |
| Often/Very often | 20 (100%) | 12 (92.3%) | 18 (94.7%) |
| **Ask your patients about their interest in quitting smoking?** |  |  |  |
| Never/Rarely/Occasionally | 2 (10.0%) | 1 (7.7%) | 2 (10.5%) |
| Often/Very often | 18 (90.0%) | 12 (92.3%) | 17 (89.5%) |
| **Talk with your patients about how to quit smoking?** |  |  |  |
| Never/Rarely/Occasionally | 0 | 1 (7.7%) | 2 (10.5%) |
| Often/Very often | 20 (100%) | 12 (92.3%) | 17 (89.5%) |
| **Recommend no pharmacotherapy?** |  |  |  |
| Never/Rarely/Occasionally | 18 (90.0%) | 8 (61.5%) | 9 (47.4%) |
| Often/Very often | 2 (10.0%) | 5 (38.5%) | 10 (52.6%) |
| **Recommend a single nicotine replacement product (e.g., patch)?** |  |  |  |
| Never/Rarely/Occasionally | 13 (65.0%) | 9 (69.2%) | 8 (42.1%) |
| Often/Very often | 7 (35.0%) | 4 (30.8%) | 11 (57.9%) |
| **Recommend dual nicotine replacement products (e.g., patch and gum or lozenge)?** |  |  |  |
| Never/Rarely/Occasionally | 15 (75.0%) | 8 (61.5%) | 11 (57.9%) |
| Often/Very often | 5 (25.0%) | 5 (38.5%) | 8 (42.1%) |
| **Recommend varenicline (Chantix)?** |  |  |  |
| Never/Rarely/Occasionally | 11 (55.0%) | 7 (53.8%) | 7 (36.8%) |
| Often/Very often | 9 (45.0%) | 6 (46.2%) | 12 (63.2%) |
| **Recommend bupropion (Zyban or Wellbutrin)?** |  |  |  |
| Never/Rarely/Occasionally | 10 (50.0%) | 10 (76.9%) | 12 (63.2%) |
| Often/Very often | 10 (50.0%) | 3 (23.1%) | 7 (36.8%) |
| **Recommend counseling to help your patients quit smoking?** |  |  |  |
| Never/Rarely/Occasionally | 11 (55.0%) | 8 (61.5%) | 9 (47.4%) |
| Often/Very often | 9 (45.0%) | 5 (38.5%) | 10 (52.6%) |
| **Arrange for a follow-up visit or phone call with your patients about quitting smoking?** |  |  |  |
| Never/Rarely/Occasionally | 15 (75.0%) | 12 (92.3%) | 14 (73.7%) |
| Often/Very often | 5 (25.0%) | 1 (7.7%) | 5 (26.3%) |
